# Supplementary material for: MicroRNAs in Small Extracellular Vesicles from Amniotic Fluid and Maternal Plasma Associated with Fetal Palate Development in Mice
Source: Int J Mol Sci. 2023 Dec 6;24(24):17173. doi: 10.3390/ijms242417173 (PMC10743272; doi:10.3390/ijms242417173)
Supplement: Supplementary file 1 [file ijms-24-17173-s001.zip › ijms-2690115-supplementary.pdf]

MicroRNAs in small extracellular vesicles from amniotic fluid and maternal plasma associated with foetal palate development in mice

Xige Zhao[1], Xia Peng[1], Zhiwei Wang[1], Xiaoyu Zheng[1], Xiaotong Wang[1], Yijia Wang[1], Jing Chen[1], Dong Yuan[2], Ying Liu[1], Juan Du[1,2*]

1 Laboratory of Orofacial Development, Laboratory of Molecular Signaling and Stem Cells Therapy, Molecular Laboratory for Gene Therapy and Tooth Regeneration, Beijing Key Laboratory of Tooth Re-generation and Function Reconstruction, Capital Medical University School of Stomatology, Tiantan Xili No.4, Beijing 100050, China

2 Department of geriatric dentistry, Capital Medical University School of Stomatology, Tiantan Xili No.4, Beijing 100050, China

* Correspondence: juandug@ccmu.edu.cn; Tel.: 86-10-57099319.

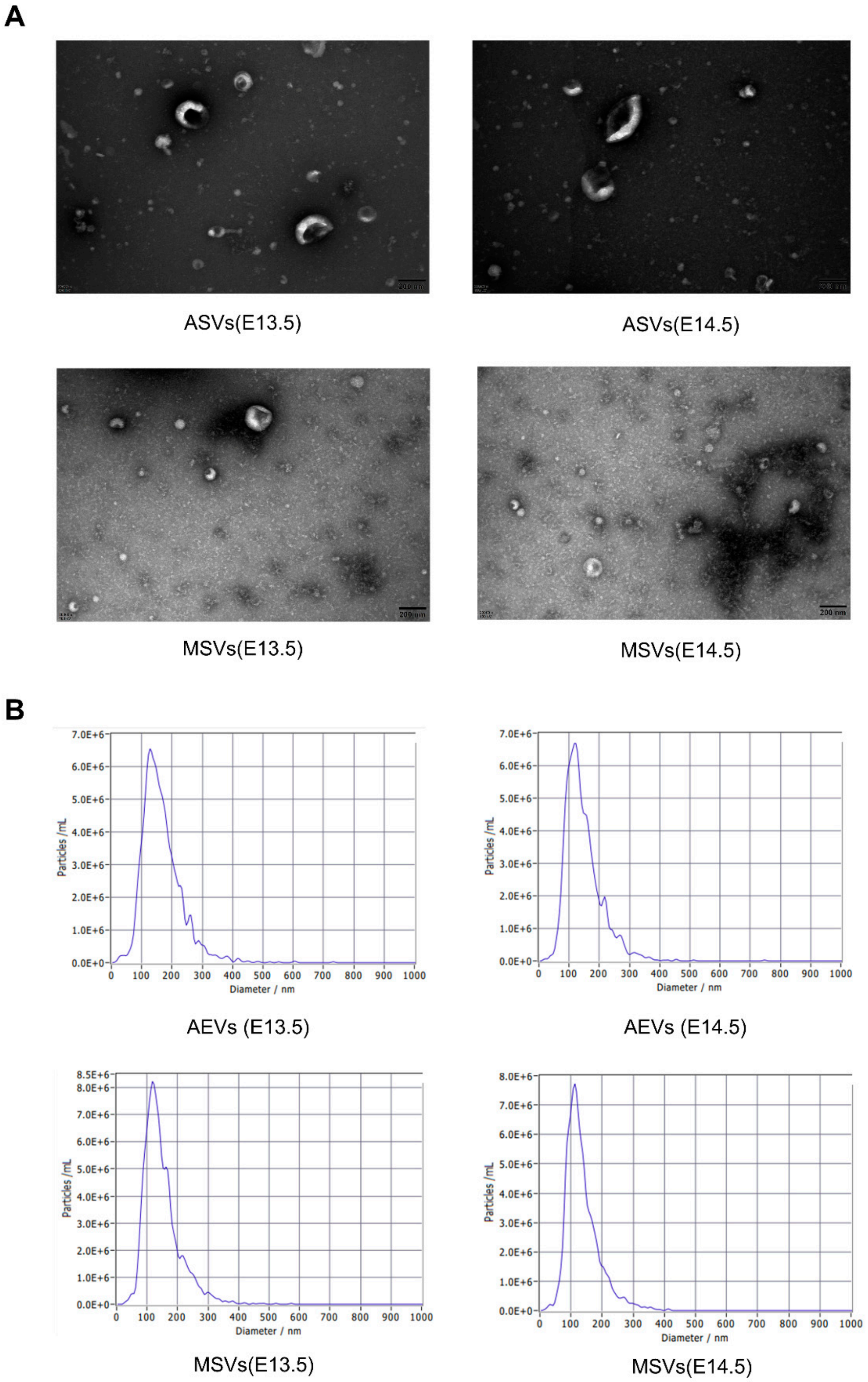

Supplemental Figure S1. Characterization of ASVs and MSVs. (**A**) TEM of ASVs (E13.5, E14.5) and MSVs (E13.5, E14.5). Scale bar, 100 nm. (**B**) NTA of ASVs (E13.5, E14.5) and MSVs (E13.5, E14.5).

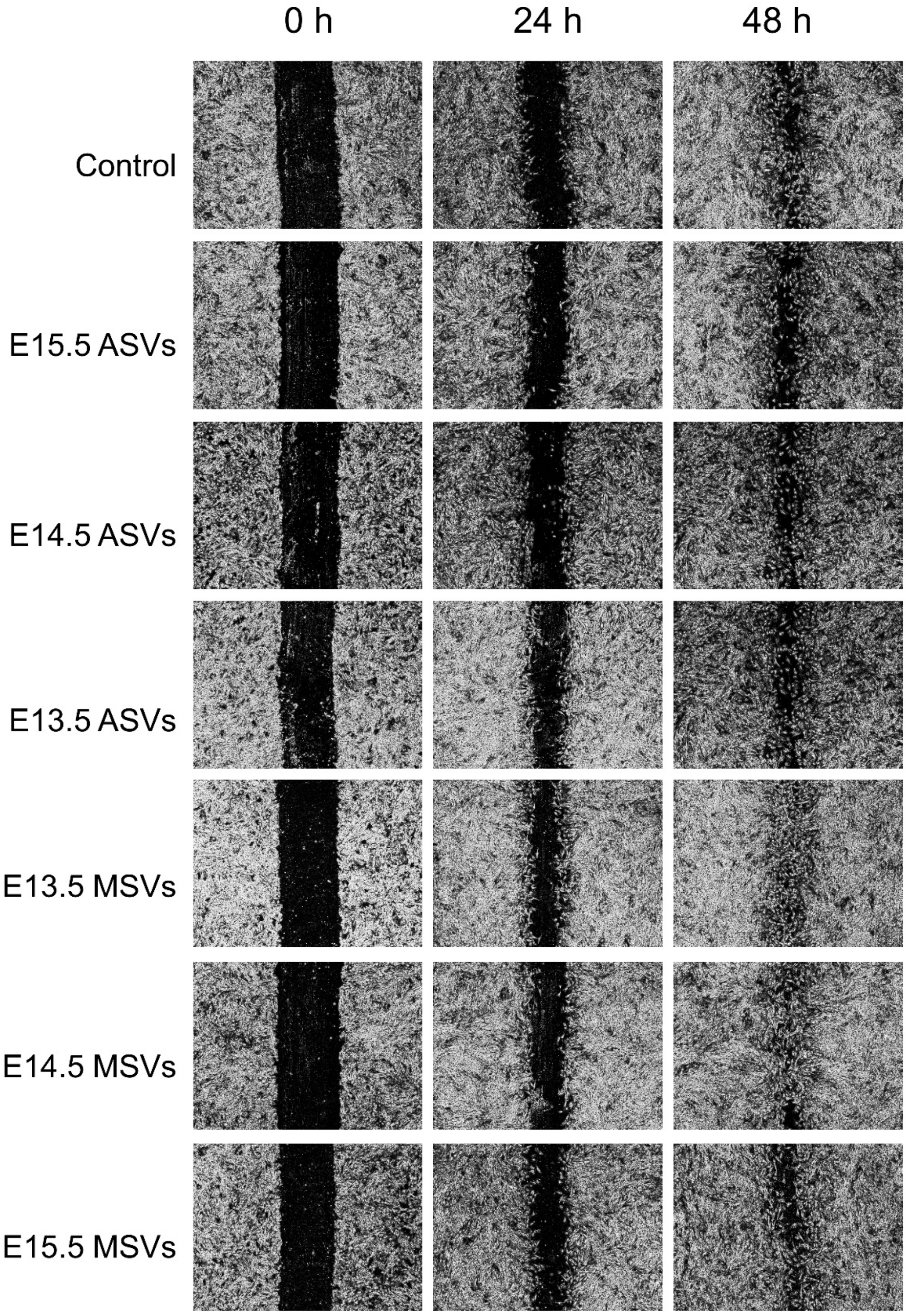

Supplemental Figure S2. Scratch assay for MEPM cells migration after treatment with different SEVs.

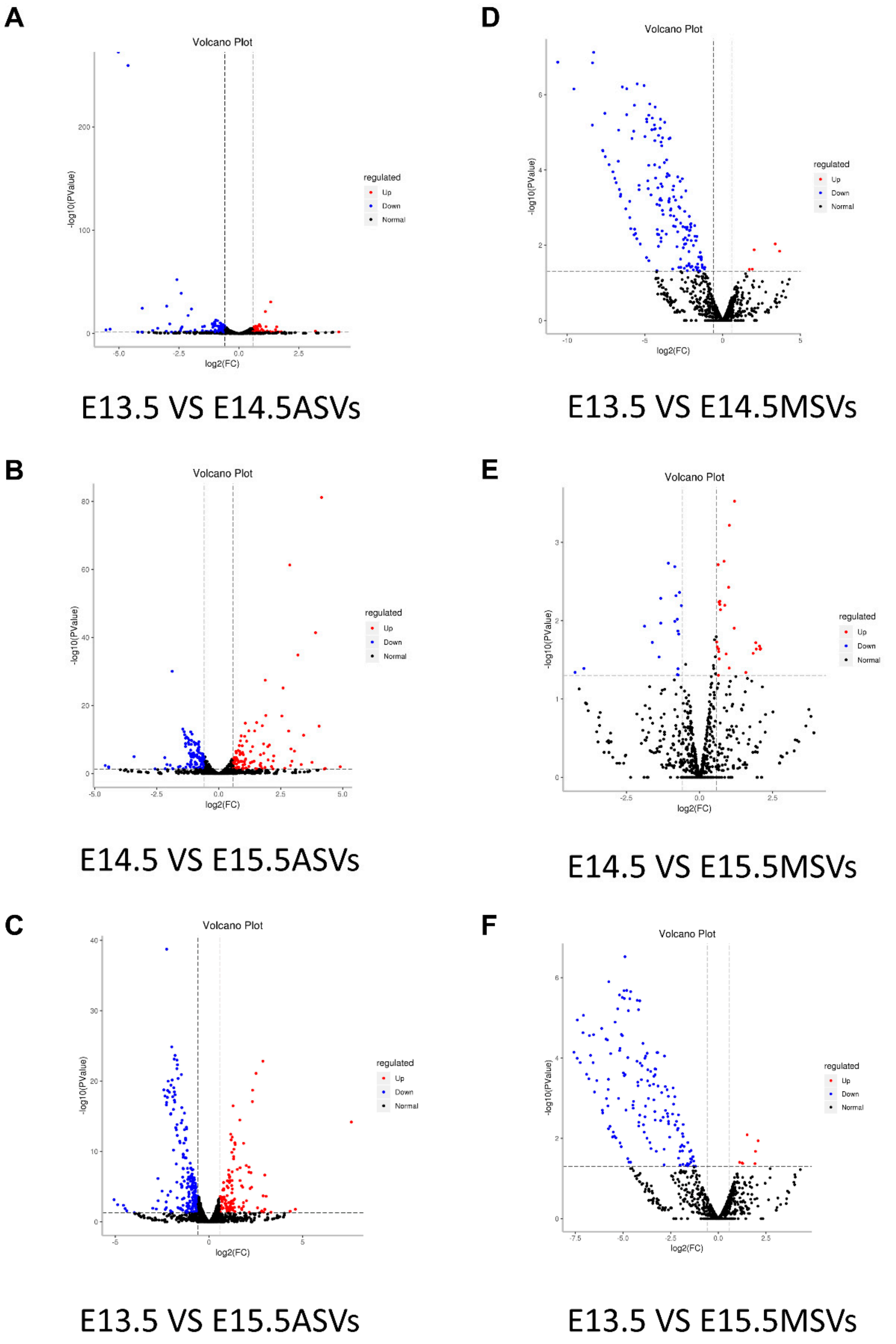

Supplemental Figure S3. Volcano plots demonstrating the fold change (*X*-axis) and P-value (*Y*-axis) of the miRNAs in ASVs or MSVs at E13.5, E14.5 and E15.5, respectively. Red and blue dots indicate the DE miRNAs, while the grey dots indicate the non-DE miRNAs.

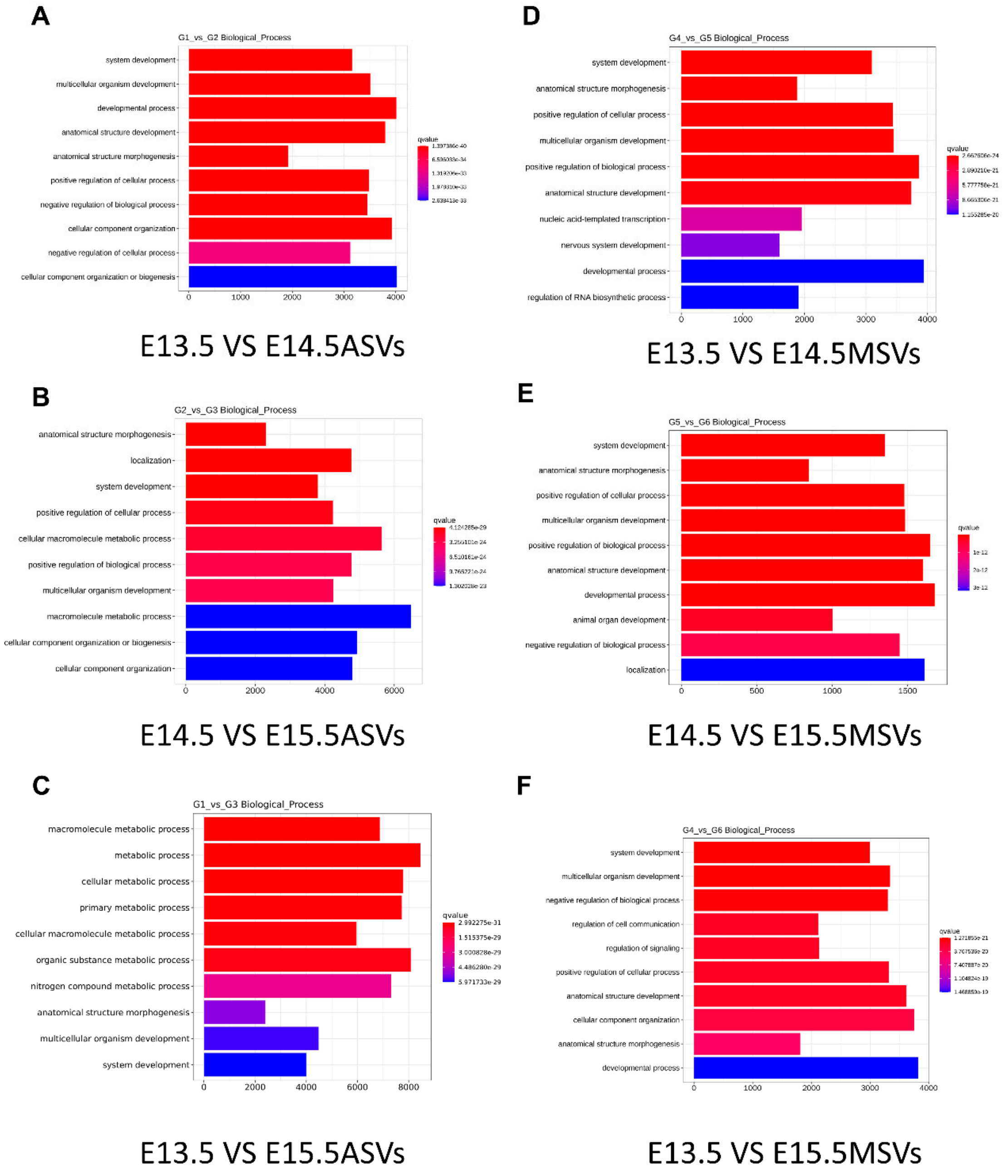

Supplemental Figure S4. Biological processes enriched in the DE miRNAs between E13.5/E14.5/E15.5 SEVs by pairwise comparison. (**A**) Bar plots depict the enriched biological processes of DE miRNAs target genes between E13.5 ASVs and E14.5 ASVs by GO assay. (**B**) Bar plots depict the enriched biological processes of DE miRNAs target genes between E14.5 ASVs and E15.5 ASVs by GO assay. (**C**) Bar plots depict the enriched biological processes of DE miRNAs target genes between E13.5 ASVs and E15.5 ASVs by GO assay. (**D**) Bar plots depict the enriched biological processes of DE miRNAs target genes between E13.5 MSVs and E14.5 MSVs by GO assay. (**E**) Bar plots depict the enriched biological processes of DE miRNAs target genes between E14.5 MSVs and E15.5 MSVs by GO assay. (**F**) Bar plots depict the enriched biological processes of DE miRNAs target genes between E13.5 MSVs and E15.5 MSVs by GO assay.

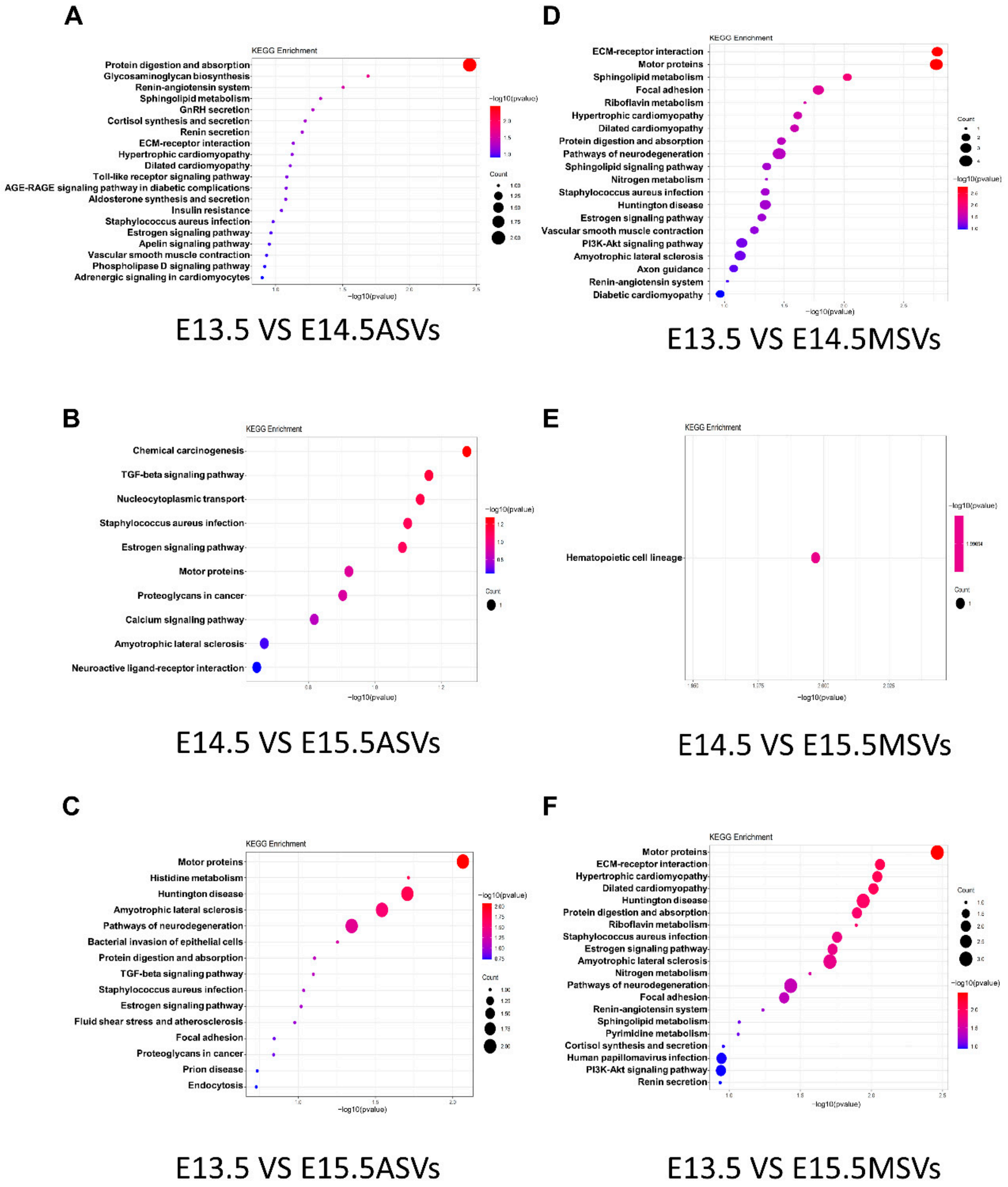

Supplemental Figure S5. Pathways enriched in the networks. (**A**) Bubble plots depict the pathways enriched in the Figure 6A regulatory network. (**B**) Bubble plots depict the pathways enriched in the Figure 6B regulatory network. (**C**) Bubble plots depict the pathways enriched in the Figure 6C regulatory network. (**D**) Bubble plots depict the pathways enriched in the Figure 7A regulatory network. (**E**) Bubble plots depict the pathways enriched in the Figure 7B regulatory network. (**F**) Bubble plots depict the pathways enriched in the Figure 7C regulatory network.

Supplemental Table S1 The primer sequences used for qRT-PCR

Primers	Sequences
miR-744-5p-F	GTGCGGGGCTAGGGCTA
miR-744-5p-R	AGTGCAGGGTCCGAGGTATT
miR-3102-5p-F	CGGTGAGTGGCCAGGGTG
miR-3102-5p-R	AGTGCAGGGTCCGAGGTATT
miR-673-5p-F	GCGCTCACAGCTCTGGTCC
miR-673-5p-R	AGTGCAGGGTCCGAGGTATT
miR-3072-3p-F	CGTGCCCCCTCCAGGAAG
miR-3072-3p-R	AGTGCAGGGTCCGAGGTATT
miR-127-3p-F	CGTCGGATCCGTCTGAGC
miR-127-3p-R	AGTGCAGGGTCCGAGGTATT
miR-298-5p-F	GGGCAGAGGAGGGCTGTT
miR-298-5p-R	AGTGCAGGGTCCGAGGTATT
U6-F	CTCGCTTCGGCAGCACA
U6-R	AACGCTTCACGAATTTGCGT
